# Microbiome-metabolomics analysis reveals abatement effects of itaconic acid on odorous compound production in Arbor Acre broilers

**DOI:** 10.1186/s12866-023-02914-w

**Published:** 2023-07-12

**Authors:** Xin Zhu, Yinhang Zhang, Haiying Liu, Guiqin Yang, Lin Li

**Affiliations:** College of Animal Science and Veterinary Medicine, Shenyang Agricultural University, Shenyang, China

**Keywords:** Skatole, Animal welfare, Itaconic acid, Bacterial community, Broiler

## Abstract

**Background:**

Public complaints concerning odor emissions from intensive livestock and poultry farms continue to grow, as nauseous odorous compounds have adverse impacts on the environment and human health. Itaconic acid is a metabolite from the citric acid cycle of the host and shows volatile odor-reducing effects during animal production operations. However, the specific role of itaconic acid in decreasing intestinal odorous compound production remains unclear. A total of 360 one-day-old chicks were randomly divided into 6 treatment groups: control group (basal diet) and itaconic acid groups (basal diet + 2, 4, 6, 8 and 10 g/kg itaconic acid). The feeding experiment lasted for 42 d.

**Results:**

Dietary itaconic acid supplementation linearly and quadratically decreased (*P* < 0.05) the cecal concentrations of indole and skatole but did not affect (*P* > 0.05) those of lactic, acetic, propionic and butyric acids. The cecal microbial shift was significant in response to 6 g/kg itaconic acid supplementation, in that the abundances of Firmicutes, *Ruminococcus* and *Clostridium* were increased (*P* < 0.05), while those of Bacteroidetes, *Escherichia-Shigella* and *Bacteroides* were decreased (*P* < 0.05), indicative of increased microbial richness and diversity. Furthermore, a total of 35 significantly (*P* < 0.05) modified metabolites were obtained by metabolomic analysis. Itaconic acid decreased (*P* < 0.05) the levels of nicotinic acid, nicotinamide, glucose-6-phosphate, fumatic acid and malic acid and increased (*P* < 0.05) 5-methoxytroptomine, dodecanoic acid and stearic acid, which are connected with the glycolytic pathway, citrate acid cycle and tryptophan metabolism. Correlation analysis indicated significant correlations between the altered cecal microbiota and metabolites; Firmicutes, *Ruminococcus* and *Clostridium* were shown to be negatively correlated with indole and skatole production, while Bacteroidetes, *Escherichia-Shigella* and *Bacteroides* were positively correlated with indole and skatole production.

**Conclusions:**

Itaconic acid decreased cecal indole and skatole levels and altered the microbiome and metabolome in favor of odorous compound reduction. These findings provide new insight into the role of itaconic acid and expand its application potential in broilers.

**Supplementary Information:**

The online version contains supplementary material available at 10.1186/s12866-023-02914-w.

## Background

A persistent tension exists between the growth of the global human population and the demand for animal food sources. To satisfy the need for meat, the quantity and scale of animal feed required are much larger than before, which leads to severe excess manure [1]. Along with manure discharge, odor emissions in commercial and intensive animal production farms are becoming an increasing vexing problem around the world, as these odorants have adverse effects on production performance, animal welfare, and the surrounding human environment [2, 3]. Therefore, sustainable methods for minimizing odor levels or inhibiting odorous compound generation urgently need to be developed.

Odorants are mainly produced by the anaerobic bacterial fermentation of nutrients, including feed residues, endogenous products and dead intestinal bacteria from the gastrointestinal tract [4]. Among these odorants, bacterially-derived tryptophan metabolites are one of the primary odor sources in farm animal feces or manure [5]. Generally, tryptophan is an essential amino acid in animals and must be supplied by the diet. Tryptophan metabolism in the gut is a multipath process that occurs in the host through a major kynurenine pathway and a minor serotonergic pathway and in the commensal microbiota through a microbial degradation pathway. The major end-product of the kynurenine pathway is nicotinamide adenine dinucleotide, along with the production of its precursors, niacin and nicotinamide, while those of the serotonergic pathway are serotonin (5-hydroxytryptamine) and melatonin. Intestinal microorganisms metabolize tryptophan into indole and its derivatives, along with tryptamine and skatole [6, 7]. Typically, the contributions of indole and skatole to the overall odor characteristics of emissions from livestock and poultry farms are very high due to their extremely low odor detection thresholds, e.g., 0.15 µg/m^3^ for indole and 3.02 µg/m^3^ for skatole [8]. Moreover, the main sites of indole and skatole production differ across the intestine. In pigs, indole and skatole are mainly generated in the colon and rectum, while in broilers, they are mainly produced in the cecum [9, 10]. Previous studies have also suggested that changes in microbial fermentation patterns are responsible for differences in intestinal indole and skatole production [11–13]. Because indole and skatole are terminal degradation products of dietary L-tryptophan by the bacterial population in the lower tracts of monogastric animals, diet is a key determinant in the production of indole and skatole; thus, reducing the availability of tryptophan and/or regulating the structure of the intestinal microbiota by dietary modification to affect anoxic metabolism of tryptophan is an essential and sustainable way of lowering or inhibiting indole and skatole production [14, 15].

Organic acids have been previously widely used to preserve feed, promote nutrient digestion and absorption, stimulate beneficial bacterial growth, and improve intestinal development in animal production [16, 17]. It has been shown that direct dietary organic acid supplementation or the indirect lowering of intestinal acidic conditions can decrease the skatole concentration of feces and plasma in pigs [18–20]. As an organic unsaturated dicarboxylic acid, itaconic acid (methylene succinic acid) is synthesized as a product of immune-responsive gene 1 (Irg1)-mediated catabolism from the citric acid cycle by decarboxylation of cis-aconitic acid in the host [21]. Many recent studies have shown that itaconic acid has multiple biological activities, such as antimicrobial, immunoregulatory, antioxidative, and nutrition-regulating activities, which have attracted increasing attention from researchers for their biological functions [22–25]. Itaconic acid can also be utilized as an active ingredient or building block in volatile odorant-reducing or contaminant-removing products to decrease the potential for odor generation or pollutant accumulation during concentrated animal feeding and wastewater treatment operations [26]. Itaconic acid is soluble in water, and the acid is generally weak but becomes stable at moderate temperatures and in acidic, neutral, and medium-basic conditions, making it a prime choice as a superabsorbent [27]. Electrostatic interactions and versatile polymerization were reported to be the chief mechanisms of itaconic acid-based materials (e.g., hydrogels, superabsorbent polymers, detergents, etc.) employed for the removal of cationic ions and pollutants from agriculture and industry [28, 29]. However, to date, there are no reports on the effects of itaconic acid on odorous compound generation from poultry farms. Therefore, the objective of this study was to investigate odorant generation and microbial community and metabolite profiles in the ceca of broilers fed diets supplemented with different levels of itaconic acid.

## Methods

### Animals and diets

A total of 360 one-day-old Arbor Acre commercial broiler chicks with similar initial live weights were used in this study. These birds were randomly allocated into 6 groups; each group contained 6 replicates, and each replicate contained 10 birds (half female and half male). Each replicate was housed in a stainless steel wire cage (length 800 mm × width 650 mm × height 400 mm) equipped with a separate feeder and a nipple drinker. The feeding room temperature was 35 ºC during the first three days and was gradually decreased by 3 ºC each week until reaching 25 ºC, and this temperature was maintained until the end of the experiment.

A corn-soybean meal basal diet without antibiotics was formulated according to China’s Feeding Standard of Chicken (NY/T33-2004, Ministry of Agriculture of the People’s Republic of China, 2004). The ingredient composition and nutrient content of the basal diet are shown in Table S1. Itaconic acid in powder form, with a purity greater than 98%, was purchased from YouQiYi Medicine Trading Co. (Fujian, China). The levels of itaconic acid added to the basal diet were 0 (Control), 2 (IA2), 4 (IA4), 6 (IA6), 8 (IA8), and 10 (IA10) g/kg. The diets were provided in an unlimited manner, and all birds were given free access to water throughout the experiment.

### Sample collection

At 42 days of the experiment, one bird with a similar live weight from each replicate was selected and then euthanized by cervical dislocation and necropsied immediately. After that, both ceca were rapidly removed, and the cecal contents were collected and mixed in a sterile tube and then frozen at -80 ºC for further analysis.

### Skatole and indole analysis

The skatole and indole analytical standards (purity ≥ 99.0%) were purchased from Sigma-Aldrich (Shanghai) Trading Co., Ltd. (Shanghai, China). A high-performance liquid chromatography (HPLC) method was used to determine the concentrations of skatole and indole in the cecal contents as described previously [11]. Briefly, 1 g of cecal contents was added to a 5-mL centrifuge tube containing 2 mL of distilled water and then homogenized with a tissue homogenizer for 15 s. After centrifugation at 3,000 × g for 10 min, 1 mL of supernatant was transferred to a 5-mL centrifuge tube containing 2 mL of methyl alcohol, mixed with a vortex mixer, and then kept at -20 ºC for 30 min to precipitate particles. After that, the tube was centrifuged at 3,000 × g for 10 min, and 1 mL of supernatant was transferred into a 1.5-mL centrifuge tube and then centrifuged at 15,000 × g for 30 min to collect the supernatant, which was then passed through a 0.45-μm filter membrane. An HPLC (1100; Agilent, Wilmington, DE) equipped with a fluorescence detector and a chromatographic column (250 mm × 4.6 mm × 5 μm; Dikma, Beijing, China) was used to analyze the concentrations of skatole and indole. A mixture of acetonitrile and distilled water at a ratio of 60:40 (v/v) was used as the mobile phase with a flow rate of 1.0 mL/min. The excitation and emission wavelengths were 263 nm and 358 nm, respectively. A volume of 20 μL of the sample was injected each time.

### Volatile fatty acid and lactic acid analyses

The concentrations of volatile fatty acids (VFAs) and lactic acid in cecal contents were determined by an HPLC system (1100; Agilent, Wilmington, DE) equipped with a photodiode array detector and a chromatographic column (250 mm × 4.6 mm × 5 μm; Dikma, China) as described previously [11]. Briefly, 1 g of cecal contents and 2 mL of distilled water were added into a 5-mL centrifuge tube, homogenized, and centrifuged at 3,000 × g for 10 min. After that, 1 mL of the supernatant liquid was transferred into a 2-mL centrifuge tube containing 0.2 mL of 25% metaphosphoric acid before centrifuging it at 15,000 × g for 10 min, after which the supernatant was collected and filtered through a 0.45-μm filter membrane. A mixture of phosphate buffer (pH = 2.5) and methyl alcohol at a ratio of 95:5 (v/v) was used as the mobile phase with a flow rate of 1.0 mL/min. A 10-μL volume of the sample was injected each time.

### DNA extraction and PCR amplification

Total DNA was extracted from the samples of the cecal contents using a commercial DNA extraction kit (Omega Bio-Tek, Guangzhou, China) according to the manufacturer’s instructions. The DNA concentration was measured by a Qubit 3.0 (Q32866; Invitrogen, Shanghai, China). The integrity of the DNA was verified by electrophoretic analysis (FR-1000, Furi Science & Technology, Shanghai, China). DNA samples were normalized to 20 ng/μL, and the amplification of 16S ribosomal DNA V3-V4 regions was conducted using universal primers (341F: CCTACGGGNGGCWGCAG; 805R: GACTACHVGGGTATCTAATCC). The amplification program of the PCR consisted of an initial denaturation at 94 ºC for 3 min, followed by 5 cycles of 94 ºC for 30 s, 45 ºC for 20 s, and 65 ºC for 30 s, 20 cycles of 94 ºC for 20 s, 55 ºC for 20 s, and 72 ºC for 30 s, and 1 cycle of 72 ºC for 5 min, followed by holding at 10 ºC until the program was terminated. The size and concentration of the amplicons were determined by 2% agarose gel electrophoresis (FR-1000, Furi Science & Technology, Shanghai, China) and Qubit 3.0 quantitation (Q32866; Invitrogen, Shanghai, China), respectively. The amplicons were pooled in equimolar concentrations to give a final concentration of 20 pM for high-throughput sequencing.

### Genomics analysis

An Illumina MiSeq high-throughput sequencing platform was used to analyze the cecal microbiota of the broilers. After sequencing, the raw reads were preprocessed by discarding the primer connector sequences using cutadapt (v 1.18) software, merging the paired reads into one sample read using PEAR (v 0.9.8) software according to the overlap between the reads, and removing the low-quality bases with a Phred score <20 using PRINSEQ (v 0.20.4) software. Whole sample reads were filtered for quality control and processed to remove non-amplified and chimeric sequences using Usearch (v 11.0.667) software to cluster operational taxonomic units (OTUs) at a threshold level of 97% sequence identity, and the sequences with the highest frequency were selected as the representative OTUs and annotated using Ribosomal Database Project Classifier (v 2.12) software and RDP 16S database. Community richness (Chao1 and ACE estimators) and diversity (Shannon and Simpson indices) were measured using Mothur (v 1.43.0) software for alpha-diversity. Similarity and/or dissimilarity between the groups were plotted for visualization using the vegan package (v 2.5-6) of R (v 3.6.0) software for principal coordinates analysis (PCoA). The dominant species in the community were displayed as heatmaps using the gplots package (v 3.0.1.1) of R (v 3.6.0) software.

### Metabolite extraction

Cecal content samples were thawed at room temperature prior to analysis. Eighty milligrams of cecal contents was weighed, and 40 μl of internal standards (L-2-chloro-phenylalanine, 0.3 mg/ml in methyl alcohol) and 360 μl of methyl alcohol were added. The mixture was adequately vortexed and homogenized at -20 °C for 5 min. Then, the homogenate was sonicated in an ice-water bath for 30 min and incubated at -20 °C for 30 min to precipitate the protein. After centrifugation at 13 000 × g and 4 °C for 10 min, 150 μl of supernatant was collected for GS-MS analysis.

### Metabolomics analysis

Chromatographic separation was performed on an Agilent GC-MS system (7890B-5977B, Agilent, Folsom, CA, USA) equipped with an HP-5MS column (30 m × 0.25 mm × 0.25 μm, Agilent, Folsom, CA, USA). The initial column oven temperature was 50 °C, which was held for 0.5 min, followed by increases to 125 °C at 8 °C/min, 210 °C at 5 °C/min, 270 °C at 10 °C/min and 305 °C at 20 °C/min, which was held for 2 min. The injection volume of each sample was 1 μl, and the carrier gas helium (purity > 99.999%) flowed at a rate of 1.0 ml/min. The inlet was set to 270 °C. The detector was operated in EI mode with filament bias at 70 eV and source and quadrupole temperatures maintained at 230 °C and 150 °C, respectively. The mode of scanning was full, and the range was 50-500 m/z. Raw data were transformed into ABF format with AnalysisBaseFileConverter software, preprocessed using MS-DIAL software and processed by the MS2Dec algorithm to qualify metabolites based on an in-house LUG library and a NIST database (https://webbook.nist.gov/chemistry/). For further quantitative analysis, the peak area of each compound was normalized to the internal standard. Then, the normalized data were subjected to principal component analysis (PCA) to obtain a visualized overview of the metabolic data, general clustering, trends, and outliers. Orthogonal partial least squares discriminant analysis (OPLS-DA) was used to determine the global alterations of metabolites between the control and IA groups. Variable importance in the projection (VIP) was calculated from the OPLS-DA model.

### Statistical analysis

The data on skatole, indole and VFAs were analyzed using one-way ANOVA via SPSS software (v 22.0, SPSS Inc., Chicago, IL), and significant differences among the groups were compared using Tukey’s multiple comparison tests. Polynomial contrasts were used to test the linear and quadratic responses to increasing levels of itaconic acid in diets. Differences between the control and itaconic acid groups (6 g/kg) in alpha-diversity, PCoA and dominant species were assessed by paired Student’s t test. Differences were considered to be significant when the probability value was less than 0.05 (*P* < 0.05). Statistically significant differences in metabolites between the control and itaconic acid groups were determined according to *P* < 0.05 and VIP > 1.0.

## Results

### Production of indole, skatole, VFAs and lactic acid

**Fig. 1:**
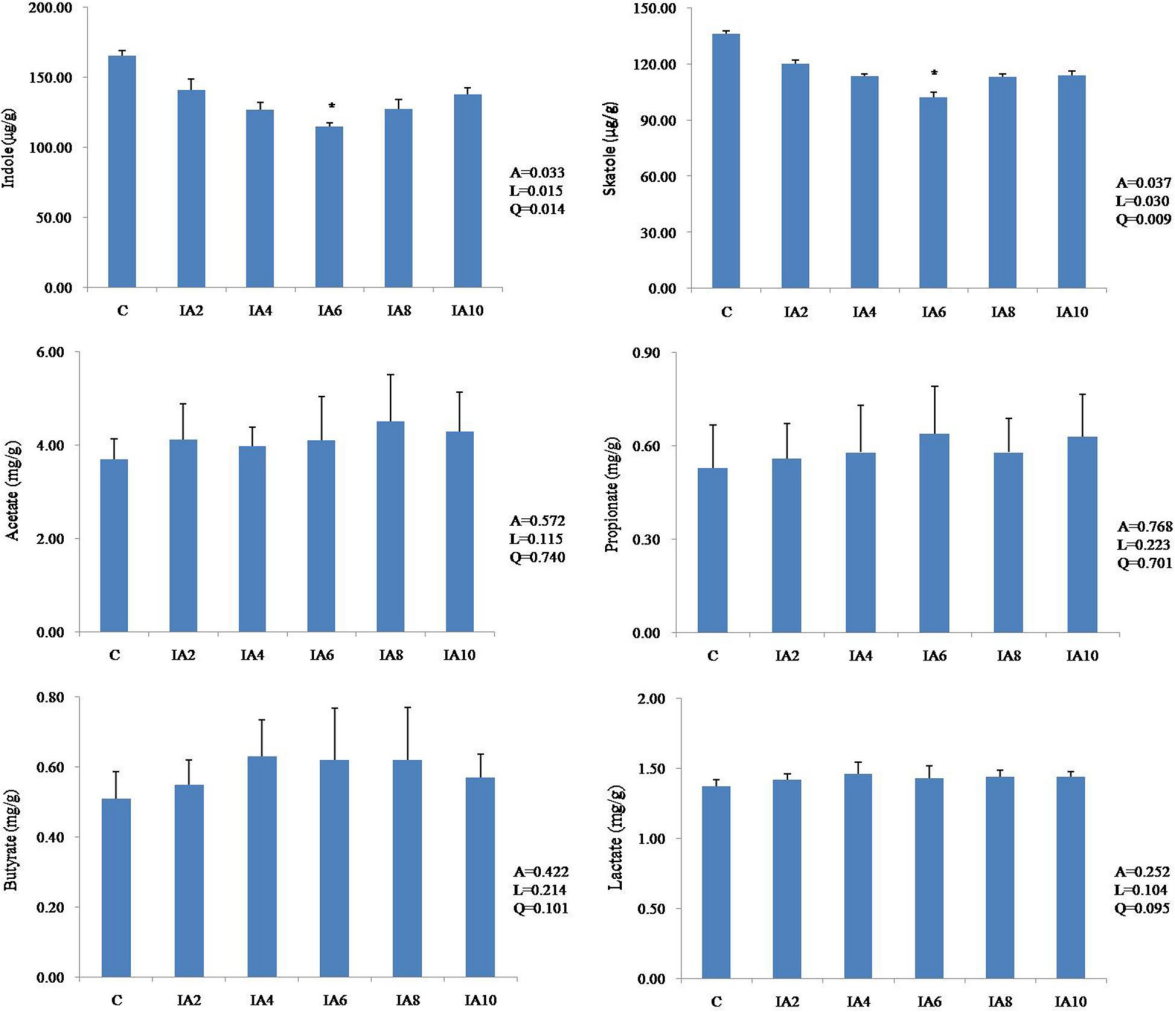
Effects of dietary itaconic acid supplementation on cecal indole, skatole, VFA and lactic acid levels in broilers at 42 days of age. C: basal diet; IA2, IA4, IA6, IA8, and IA10 groups: basal diet supplemented with 2,4,6,8, and 10 g/kg itaconic acid, respectively. * represents a significant difference between the control and IA groups, *P*< 0.05. A: *P* value of one-way ANOVA; L, *P* value of linear analysis; Q, *P* value of quadratic analysis

As shown in Fig. 1, dietary itaconic acid supplementation had a significant effect on the concentrations of indole and skatole in the cecum of broilers (*P* < 0.05). In addition, both linear and quadratic effects on indole and skatole concentrations were observed with increasing levels of itaconic acid (*P* < 0.05). Compared with the control, the IA6 group had the lowest concentrations of indole and skatole (*P* < 0.05), but there were no significant differences among the other itaconic acid groups (*P* > 0.05). Notably, no significant difference was observed in the VFA and lactic acid concentrations among all groups (*P* > 0.05).

### Microbial richness and diversity

**Fig. 2:**
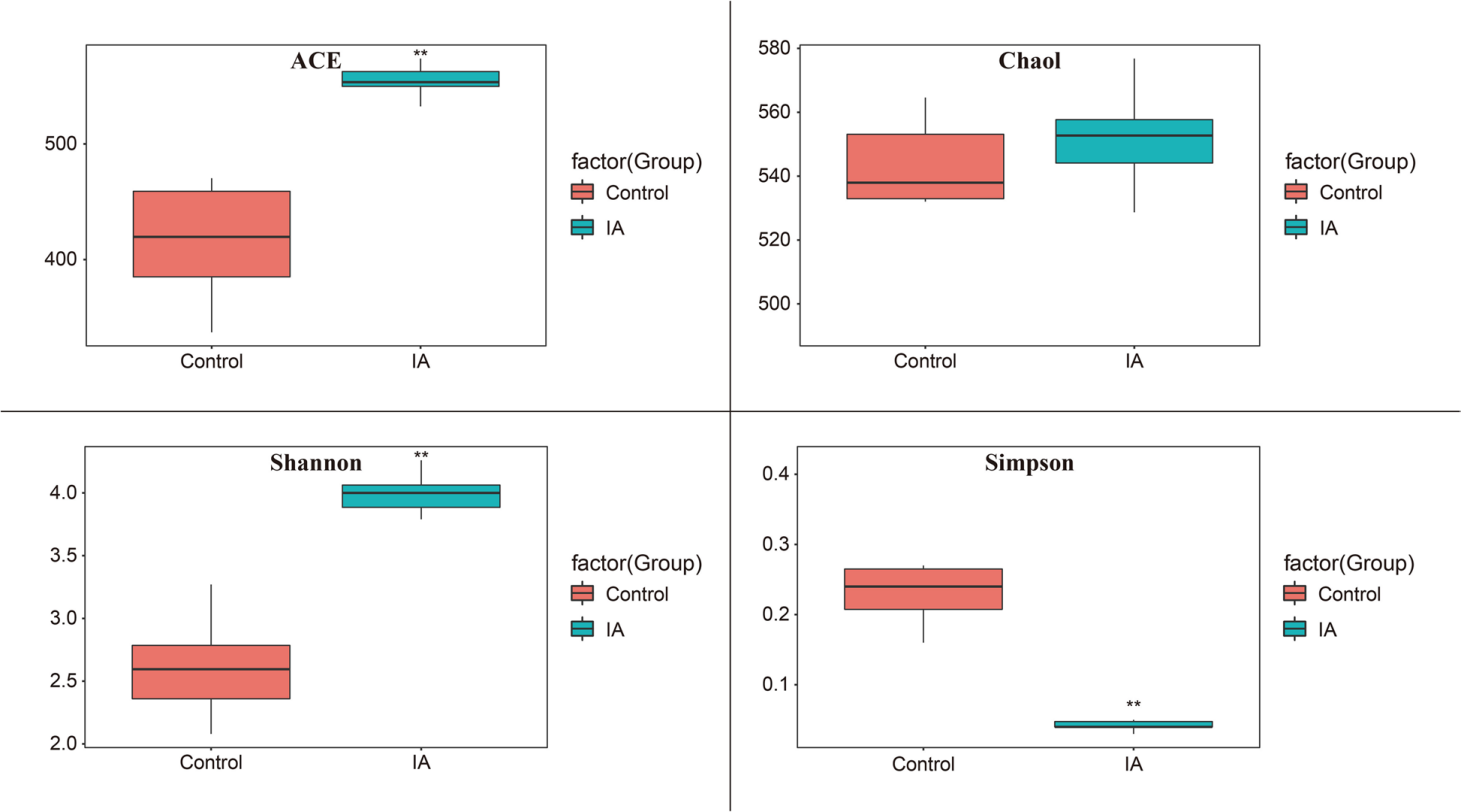
Alpha diversity of the microbial community in the cecal digesta of broilers at 42 days of age. Control: basal diet; IA group: basal diet supplemented with 6 g/kg itaconic acid

Because the IA6 group showed the lowest concentrations of indole and skatole, this group was selected for further microbiome and metabolome analyses. After filtering the sequences, the control and itaconic acid groups had 412,450 and 517,004 reads, respectively, and the sequence lengths were 419 and 412 bp after quality control (removal of the barcode, primer, and low-quality sequences), respectively. A total of 553 and 659 OTUs were identified in cecal samples from the control and itaconic acid groups, respectively, in which the numbers of OTUs specific for the control and IA6 groups were 22 and 128, respectively. The number of shared OTUs between the two groups was 531. The alpha diversity analysis in the two groups revealed that the ACE and Shannon indices of the itaconic acid group were significantly higher than those in the control group (*P* < 0.01), suggesting that dietary itaconic acid supplementation increased the richness and diversity of the cecal microbiota (Fig. 2).

### Microbiota composition

**Fig. 3:**
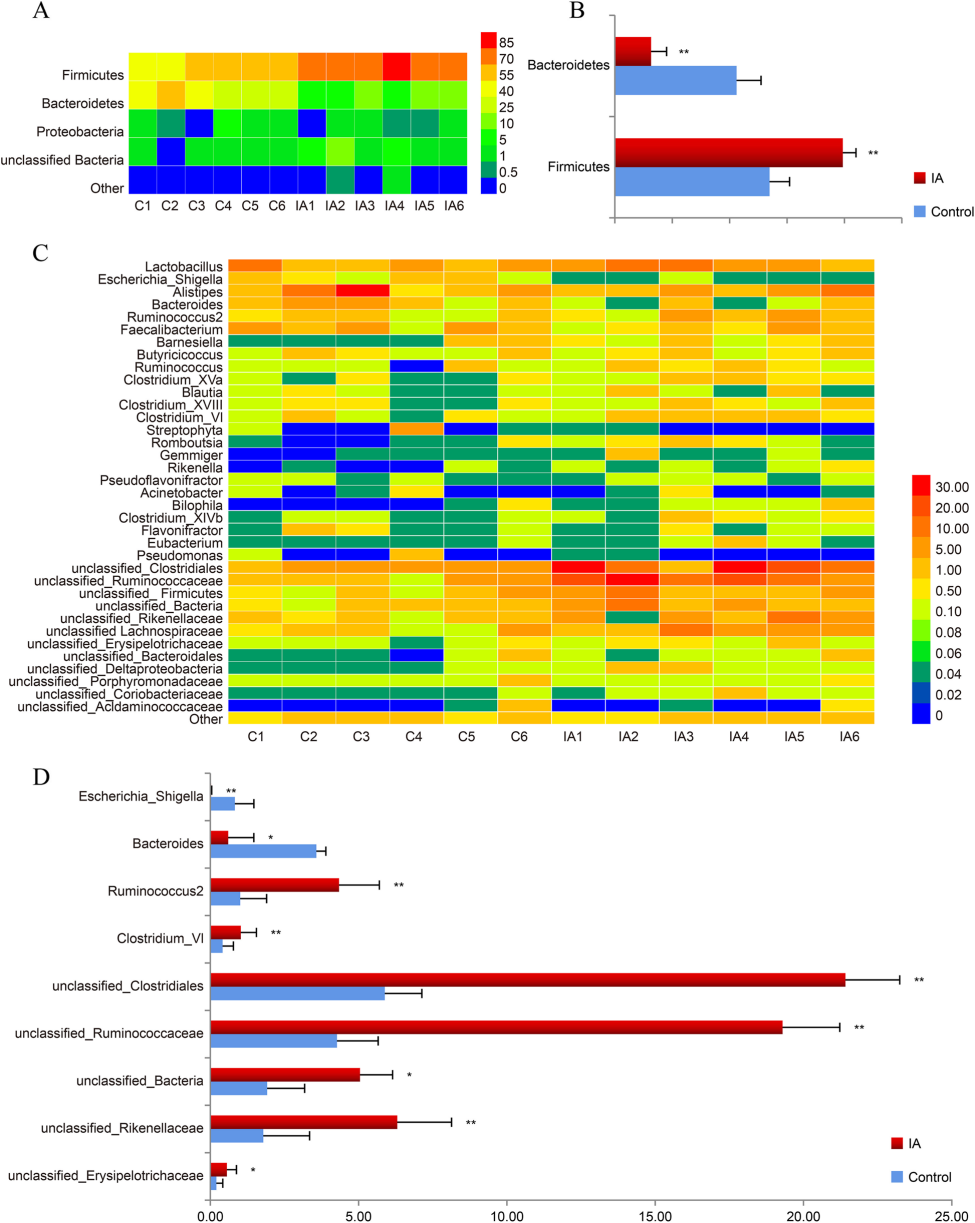
Cecal microbial community structure at the phylum (**A**) and genus (**C**) levels (*n* = 6) in broilers at 42 days of age. The taxa with higher abundances in the groups are shown in red, while those with lower abundances are shown in blue. The significant changes in phyla (**B**) and genera (**D**) are presented. The values are expressed as the medians. Statistical differences were calculated by paired Student’s t test. * represents *P* < 0.05 and ** represents *P* < 0.01. C: basal diet; IA group, basal diet supplemented with 6 g/kg itaconic acid

At the phylum level, a total of 15 phyla and 1 unclassified bacteria were identified in all samples, but only three phyla and one unclassified bacteria were abundant by more than 1%, and their average relative abundances were over 99.5% of the overall bacterial community. Firmicutes, Bacteroidetes, Proteobacteria and unclassified bacteria were the four most abundant bacteria, with abundance ranges of 40.56-85.52%, 6.66-58.35%, 0.16-5.11% and 0.18-10.50%, respectively (Fig. 3A). The relative abundance of Firmicutes and unclassified bacteria in the itaconic acid group was significantly higher than that in the control group (*P* < 0.05), while the relative abundance of Bacteroidetes was significantly lower (*P* < 0.05). There was no significant difference in the relative abundance of Proteobacteria between the control and itaconic acid groups (*P* > 0.05) (Fig. 3B).

At the genus level, a total of 123 genera were identified in all samples, but only 36 genera showed relative abundances greater than 1% (Fig. 3C). The dominant bacteria between the two groups were quite different. Among the top five genera from the control, the relative abundance of *Alistipes* was the highest (10.01%), followed by *unclassified Clostridiales* (5.88%), *Lactobacillus* (5.41%), unclassified *Ruminococcaceae* (4.27%) and *Faecalibacterium* (3.66%). In the itaconic acid group, the top five most abundant genera were unclassified *Clostridiales* (21.41%), unclassified *Ruminococcaceae* (19.29%), *Lactobacillus* (8.02%), *Alistipes* (6.91%) and unclassified *Firmicutes* (6.70%). The results also showed that *Ruminococcus2*, *Clostridium XIVa*, unclassified *Clostridiales*, *Ruminococcaceae*, *Bacteria*, *Rikenellaceae* and *Erysipelotrichaceae* were more abundant in the itaconic acid group (*P* < 0.05), while the relative abundances of *Escherichia-Shigella* and *Bacteroides* were higher in the control group (*P* < 0.05) (Fig. 3D).

### Metabolomic profiles

**Fig. 4:**
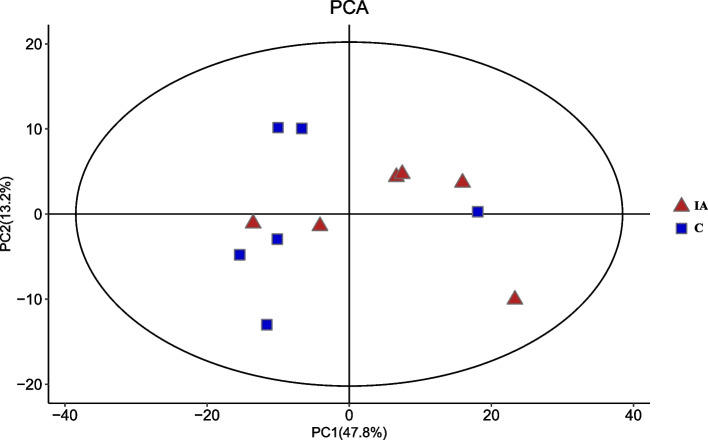
Principal component analysis (PCA) of cecal metabolites from all the samples in broilers at 42 days of age. C: basal diet; IA group: basal diet supplemented with 6 g/kg itaconic acid

**Fig. 5:**
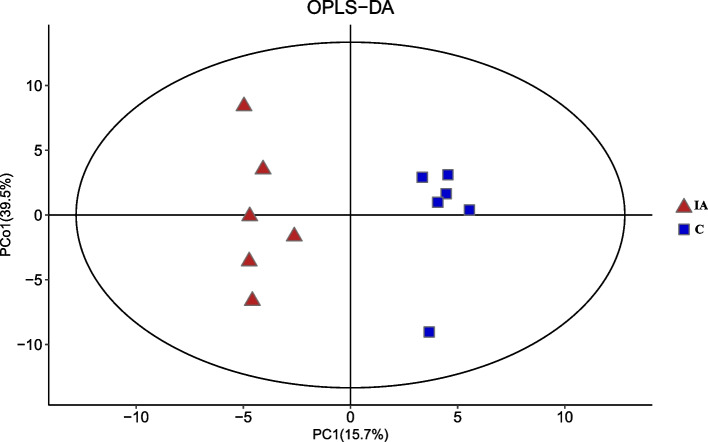
Orthogonal partial least squares discriminate analysis (OPLS-DA) score plots of cecal metabolites from all the samples in broilers at 42 days of age. A more separated distribution of the samples represents a lower pattern of similarity, and vice versa. C: basal diet; IA group: basal diet supplemented with 6 g/kg itaconic acid

The PCA results showed that the samples clustered closely, suggesting that a variety of metabolites were identified in both the control and itaconic acid groups (Fig. 4). OPLS-DA score plots further revealed a remarkable separation of these two groups (Fig. 5).

**Fig. 6:**
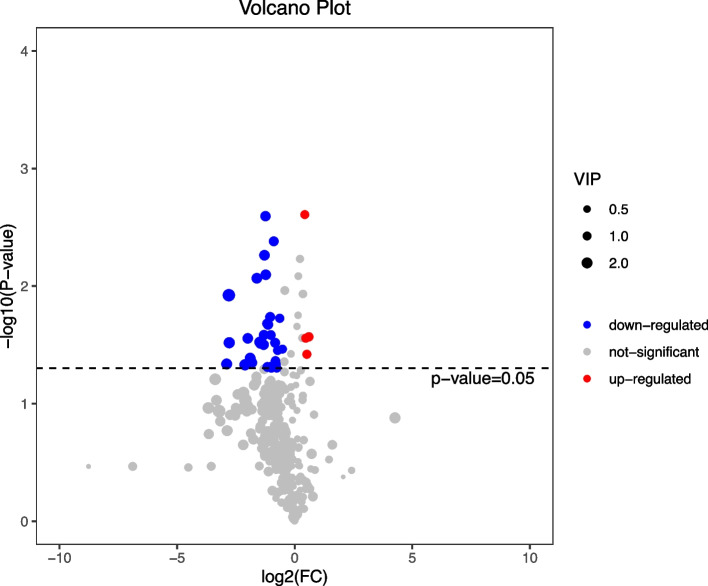
Volcano plot of metabolites with significant differences between the control and itaconic acid groups in broilers at 42 days of age (*n* = 6). The size of the circles (VIP values) indicates the extent of the contribution of the marker metabolite to the difference between the control and itaconic acid groups. Broilers in the control group were fed a basal diet, and broilers in the itaconic acid group were fed a basal diet supplemented with 6 g/kg itaconic acid

**Fig. 7:**
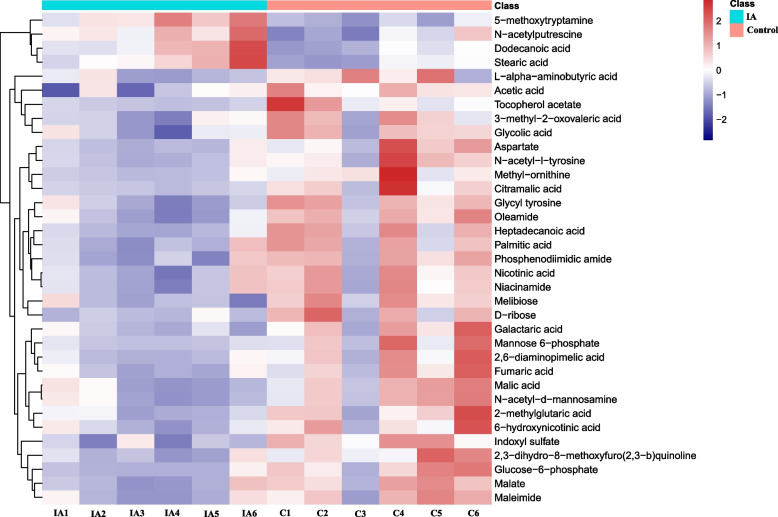
Heatmap visualizing the significantly altered metabolites in the cecal digesta of broilers at 42 days of age (*n* = 6). C: basal diet; IA group: basal diet supplemented with 6 g/kg itaconic acid. Red and blue colors represent high and low levels of metabolites, respectively

In total, 388 metabolites were identified in both groups. After t test and VIP filtering, 35 metabolites were found to be significantly changed between the control and itaconic acid groups (*P* <0.05), among which 31 metabolites were significantly decreased and 4 metabolites were increased in the itaconic acid group compared with the control (*P* < 0.05) (Fig. 6). In addition, 13 metabolites belonged to the superclass of organic acids and derivatives, and 8 metabolites belonged to the superclass of lipids and lipid-like molecules. Notably, compared to the control, 3 metabolites that belonged to the class of pyridines and derivatives in the itaconic acid group were significantly decreased (*P* < 0.05) (Fig. 7).

### Correlations of the cecal microbiome and metabolome

**Fig. 8:**
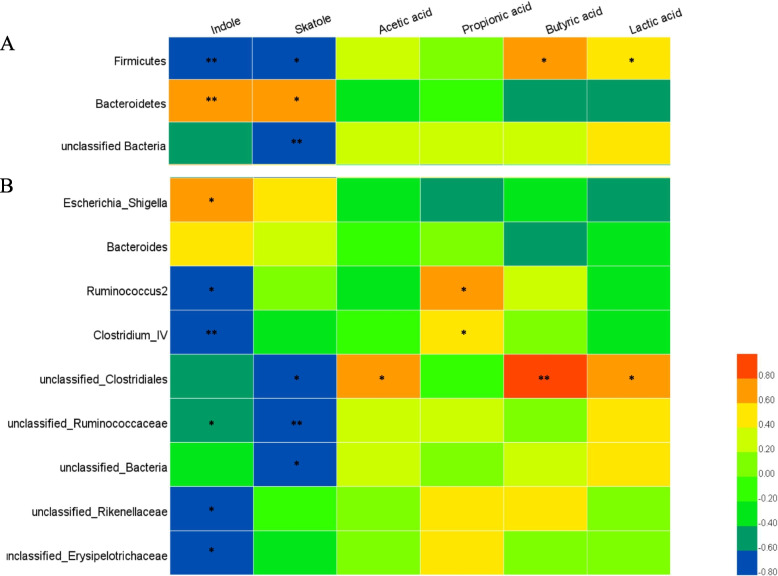
Correlation analysis between the discriminated cecal microbiota and the odorous compounds in broilers at 42 days of age (*n* = 6). A: at the phylum level; B: at the genus level. Red indicates a positive correlation, and blue indicates a negative correlation. * represents *P* < 0.05 and ** represents *P* < 0.01

To reveal relationships between the cecal microbiota and odorous compounds, Pearson’s correlation analysis was performed. As shown in Fig. 8A, at the phylum level, the relative abundance of Firmicutes was negatively correlated with the indole and skatole levels, while that of Bacteroidetes was positively correlated with the indole and skatole levels. At the genus level, the abundances of *Ruminococcus2*, *Clostridium IV*, unclassified *Ruminococcaceae*, *Rikenellaceae* and *Erysipelotrichaceae* were negatively correlated with the level of indole, while that of *Escherichia-Shigella* was positively correlated with the level of indole. We also found that the abundances of unclassified *Clostridiales* and *Ruminococcaceae* were negatively correlated with the level of skatole (Fig. 8B).

**Fig. 9:**
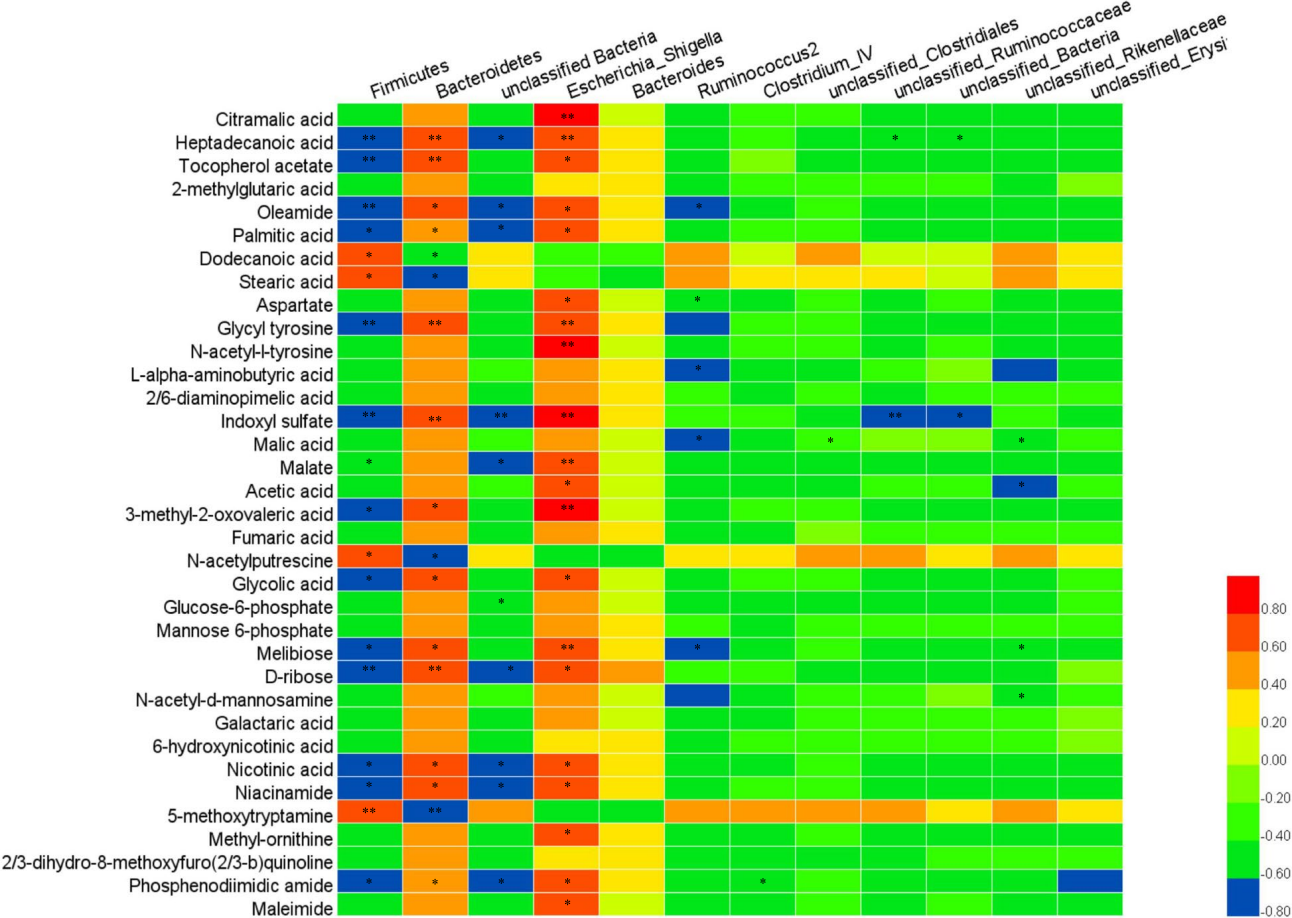
Correlation analysis between the altered cecal microbiota and metabolites in broilers at 42 days of age (*n* = 6). Red indicates a positive correlation, and blue indicates a negative correlation. * represents *P* < 0.05 and ** represents *P* < 0.01

Pearson’s correlation analysis of the functional correlations between the changes in the cecal microbiome and the metabolome was also conducted. As shown in Fig. 9, at the phylum level, a total of 43 significant correlations were recognized, among which 17 correlations were positive and 26 correlations were negative. Specifically, Firmicutes mainly had 4 negative correlations with lipids and lipid-like molecules, 4 negative correlations with organic acids and derivatives, 2 negative correlations with organic oxygen compounds and 2 negative correlations with organoheterocyclic compounds; in contrast, Bacteroidetes mainly had positive correlations with these compounds. Similar to Firmicutes, unclassified bacteria had more negative correlations with altered metabolites identified between the control and itaconic acid groups. At the genus level, a total of 42 significant correlations were recognized, among which 20 correlations were positive and 12 correlations were negative. Interestingly, all 20 positive correlations were found in *Escherichia-Shigella*, while other negative correlations were mainly found in *Ruminococcus2* and unclassified *Rikenellaceae*.

## Discussion

In this study, we demonstrated that supplementation with itaconic acid in the diet decreased the concentrations of indole and skatole in the cecum of broilers, indicating that organic acids may be another choice for reducing odorous compound production. Similarly, the results from Claus et al. (2003) and Øverland et al. (2008) have shown that the addition of organic acids to the diet can decrease indole and skatole formation in pigs [18, 20]. To date, because of the variety of functional groups, itaconic acid can be converted to a number of high-value biobased chemicals or materials, such as porous hydrogels, which are used for the removal of contaminants from the environment [27]. However, to date, there are no reports about the efficacy of itaconic acid in reducing odorous compound production in livestock and poultry industries. Presumably, in this study, the addition of itaconic acid to the diet created an acidic environment in the gut that favored the growth of beneficial bacteria and inhibited the replication of pathogenic and indole-metabolizing bacteria, thus reducing the production of indole and skatole and other microbial L-tryptophan metabolites. VFAs, which are also known as short-chain fatty acids (SCFAs), are produced mainly from anaerobic microbial fermentation of dietary indigestible carbohydrates in the lower digestive tracts of monogastric animals, along with the formation of a few other organic compounds, such as lactic acid. Dietary supplementation with organic acids can lower stomach pH and increase digestive enzyme activity, but it has little effect on the pH of the lower intestinal tract and VFA concentrations [30]. Our results showed that dietary supplementation with itaconic acid did not affect the VFA and lactic acid concentrations of the cecum in broilers.

In general, the predominant phyla in the avian gastrointestinal tract are Firmicutes, Bacteroidetes, Proteobacteria, Actinobacteria, and Tenericutes [31]. Similarly, the results from the present study also showed that Firmicutes, Bacteroidetes and Proteobacteria represented the majority of the cecal microbiota in the broilers. Bacteria in Firmicutes and Bacteroidetes are used as phylogenetic markers since their abundance is easily influenced by the fermentation condition of the gut [4]. *Bacteroides* and *Alistipes* were demonstrated to metabolize tryptophan to indole-3-lactate and then to indole and skatole [11], indicating that the higher abundance of *Bacteroides* and *Alistipes* was usually associated with the higher concentrations of skatole and indole in the gut. *Ruminococcus* and *Clostridium* are important VFA producers in the gut, and an increase in these bacteria may compete with the growth of indole- and skatole- producing bacteria, thus lowering the concentration of odorous compounds. *Lactobacillus* can produce lactic acid, which has been reported to reduce gastric pH and delay the proliferation of pathogenic bacteria such as *E. coli* and to be widely used in animal feed to enhance gut health and production performance [32, 33]. *Rikenellaceae* has the ability to ferment carbohydrates to produce acetic acid and has also been negatively associated with inflammation and impairment of mucosal immune functions [34]. *Erysipelotrichaceae* was previously found to be strongly positively correlated with tryptophan levels in the intestinal tract and the feed conversion ratio performance of broilers, indicative of regulation of amino acid metabolism and body weight gain [35, 36]. The present study indicated that dietary supplementation with itaconic acid led to a higher richness and diversity of the cecal microbiota, increased abundance of *Ruminococcus*, *Clostridium*, *Rikenellaceae* and *Erysipelotrichaceae* and decreased abundance of *Bacteroides*, thus lowering the cecal concentrations of indole and skatole in broilers.

Our metabolomic data showed that feeding broiler chickens an itaconic acid diet produced a number of varied metabolites related to nicotinate and nicotinamide, pyruvate, fatty acid biosynthesis and citrate acid cycle metabolism. Nicotinic acid and nicotinamide (members of the vitamin B_3_ family) are essential and must be supplied by the diet in animals unable to synthesize them, as they are precursors for the coenzymes NAD and NADP *in vivo*, which participate in many cellular pathways, including carbohydrate, lipid and protein metabolism [37]. Many bacteria can utilize nicotinic acid as a carbon and nitrogen source and degrade it under aerobic or anaerobic conditions. 6-Hydroxynicotinic acid is formed in the first step of the degradation of nicotinic acid and is then transformed into maleic acid, fumatic acid and pyruvic acid through an aerobic pathway and into propionic acid, acetic acid and pyruvic acid through an anaerobic pathway [38]. Nicotinic acid and nicotinamide are mainly produced from the kynurenine pathway of dietary tryptophan metabolism [39]. Observations from this study showed a decrease in nicotinate and its metabolites in the cecal contents, indicating that dietary supplementation with itaconic acid may change the flux of tryptophan to be used by the host or intestinal microbes. In addition to nicotinic acid production, tryptophan can also be metabolized into serotonin (5-hydroxyltrptamine) through the serotonin pathway; serotonin is not only a neurotransmitter with multiple functions in the central nervous system but also regulates intestinal movements in the gastrointestinal tract [40]. The majority of serotonin in the host (~95%) is produced by enterochromaffin cells in the gut and is released into the gut lumen in response to various mechanical and chemical stimuli [41]. Serotonin can be converted into melatonin, which is involved in the regulation of circadian rhythms, food intake and digestion [42], by subsequent acetylation and methylation, and melatonin can be further metabolized into 5-methoxytryptamine by deacetylation [43]. The administration of L-tryptophan (150-300 mg/kg) to rats and chicks was previously demonstrated to cause a rapid and dose-dependent elevation of circulating melatonin [44]. Interestingly, the results from this study showed that the cecal 5-methoxytryptamine concentration was increased after dietary itaconic acid supplementation, indicating that itaconic acid may have a regulating activity favoring the serotonin pathway of tryptophan in the gut, in which gut motility, feed intake and nutrient digestion were improved, and have an effect of nutrient repartition in which less tryptophan was available for microbial degradation into indole and skatole in the ceca of broiler chickens.

Itaconic acid has also been demonstrated to be a key regulator of the glycolytic pathway and the citrate acid cycle, which is related to energy metabolism [45]. Itaconic acid was previously found to be an inhibitor of phosphofructokinase 2, which catalyzes the phosphorylation of fructose-6-phosphate to fructose-2,6-bisphosphate, and the latter in turn allosterically activates phosphofructokinase 1, which is a rate-limiting enzyme and catalyzes the phosphorylation of fructose-6-phosphate to fructose-1,6-biphosphate [46]. Due to this inhibitory effect on the glycolytic pathway, itaconic acid may decrease the production of pyruvic acid, an end product of the glycolytic pathway, and suppress the synthesis of fatty acids from glucose [21]. As a decarboxylation product from cis-aconitic acid, a citrate acid cycle intermediate, itaconic acid has an inhibitory effect on succinate dehydrogenase (SDH), leading to the accumulation of succinic acid, thus resulting in decreased fumaric acid and malic acid formation in the citrate acid cycle [45]. The results from this study showed that metabolites from pyruvate metabolism and the citrate acid cycle, including glucose-6-phosphate, fumaric acid and malic acid, were decreased after dietary itaconic acid supplementation, indicative of a negative regulatory effect on energy metabolism. For fatty acid metabolism, the results from our study showed that the concentration of palmitic acid was decreased, while those of dodecanoic acid and stearic acid were increased. Considering the inhibitory effect of itaconic acid on the glycolytic pathway, it is reasonable to infer that itaconic acid may have an inhibitory influence on fat synthesis. Recently, the results from Zhu et al. (2022) showed that dietary supplementation with 0.69% itaconic acid decreased the abdominal fat yield of broilers at 42 days of age [47]. However, the effect and mechanism of itaconic acid on lipid metabolism should be studied further.

A bidirectional association between the metabolome and the microbiome in the gut exists and is fundamental for poultry production and health [48]. Significant correlations were observed between the altered cecal metabolites and microbiota, such as Firmicutes and Bacteroidetes. As dominant phyla in the gut, Firmicutes and Bacteroidetes always coexist and even show some aspects of cooperation or specialization, although an increase in one phylum corresponds to a decrease in the other [49, 50]. Bacteroidetes has been described as a saccharolytic taxon that produces primarily propionate, while Firmicutes tends to be efficient in proteolytic, saccharolytic and lipolytic digestion in equal measure, mainly producing metabolites that are related to amino acid, glucose and lipid metabolism [51]. The results from this study showed that both Firmicutes and Bacteroidetes had varied, even opposite, relationships with cecal metabolite profiles, indicating that these phyla may have different regulatory effects on gut homeostasis. Of note, at the genus level, in this study, *Escherichia-Shigella* showed positive correlations with lipid metabolites (such as palmitic acid and heptadecanoic acid), organic acids (such as malic acid and acetic acid) and organoheterocyclic compounds (such as nicotinic acid and niacinamide), while *Ruminococcus2* and unclassified *Rikenellaceae* showed negative correlations with these metabolites. All these correlations indicated that dietary itaconic acid can affect the cecal microbiota and its associated altered metabolites that might be involved in odorous compound production, which is worth further exploration.

## Conclusions

The present study combining microbiome and metabolome analyses demonstrated that dietary itaconic acid supplementation selectively modulated the cecal microbiota composition and metabolic activity to lower the production of odorous compounds indole and skatole in broilers. Cecal bacteria *Ruminococcus*, *Clostridium*, *Rikenellaceae* and *Erysipelotrichaceae* increased in abundance and *Bacteroides* decreased in abundance after feeding an itaconic acid diet. The cecal metabolites were changed by dietary itaconic acid supplementation, as evidenced by the decrease in the levels of nicotinic acid, nicotinamide, glucose-6-phosphate, fumatic acid and malic acid and increase in the levels of 5-methoxytroptomine, dodecanoic acid and stearic acid, which are connected with the glycolytic pathway, citrate acid cycle and tryptophan metabolism. To the best of our knowledge, this research is the first to shed light on the odor-lowering activity of itaconic acid in broilers and to comprehensively demonstrate the underlying mechanism. These results may be helpful in developing novel odorous compound-lowering strategies in food animal production.

## Supplementary Information

**Additional file 1: Table S1. **Composition and nutrient levels of the basal diet (air-dry basis, g/kg).

## Data Availability

All data and materials are available in the manuscript and as supplementary. The 16S rRNA gene sequences determined in this study were deposited in the NCBI BioProject database (https://www.ncbi.nlm.nih.gov/sra/PRJNA955893 ; Accession number: PRJNA955893).

## Abbreviations

ANOVA: Analysis of variance
HPLC: High-performance liquid chromatography
IAA: Indoleacetic acid
GS-MS: Gas chromatography-mass spectrometry
OUT: Operational taxonomic unit
PCoA: Principal coordinates analysis
SCFA: Short chain fatty acid
VFA: Volatile fatty acid
VIP: Variable importance in the projection
