# Supplementary material for: Microbiome-metabolomics analysis reveals abatement effects of itaconic acid on odorous compound production in Arbor Acre broilers
Source: BMC Microbiol. 2023 Jul 12;23:183. doi: 10.1186/s12866-023-02914-w (PMC10337203; doi:10.1186/s12866-023-02914-w)
Supplement: Supplementary file 1 — Additional file 1: Table S1. Composition and nutrient levels of the basal diet (air-dry basis, g/kg). [file 12866_2023_2914_MOESM1_ESM.docx]

**Table S1**

Composition and nutrient levels of the basal diet (air-dry basis, g/kg)

| Items | Content | | | | | |
| --- | --- | --- | --- | --- | --- | --- |
|  | d 1 to 21 | d 22 to 42 | |  | d 1 to 21 | d 22 to 42 |
| Ingredients |  |  | Analyzed values | | | |
| Corn | 619 | 600 | Crude protein | | 211.9 | 204.9 |
| Soybean meal | 280 | 276 | Crude fat | | 32.4 | 61.3 |
| Corn gluten meal | 28 | 35 | Calcium | | 9.9 | 7.5 |
| Soybean oil | 8 | 35 | Total phosphorus | | 8.9 | 6.4 |
| Monosodium glutamate residue | 15 | 15 | Calculated composition | | | |
| Limestone | 15.0 | 10.0 | Metabolizable energy (MJ/kg) | | 12.15 | 12.98 |
| Calcium hydrophosphate | 14.0 | 10.0 | Available phosphorus | | 3.7 | 3.0 |
| Sodium chloride | 3.0 | 2.5 | Lysine | | 13.4 | 12.8 |
| Choline chloride | 1.0 | 1.0 | Methionine | | 5.0 | 5.1 |
| L-lysine | 6.5 | 6.5 | Threonine | | 10.0 | 8.3 |
| DL-methionine | 1.8 | 2.0 | Tryptophan | | 2.8 | 2.1 |
| L-threonine | 2.3 | 1.0 |  | |  |  |
| Premix^1^ | 6.0 | 6.0 |  | |  |  |

^1^ The premix provided per kg of diets: Cu (as copper sulfate), 16 mg; Fe (as ferrous sulfate), 180 mg; Mn (as manganese sulfate), 87 mg; Zn (as zinc sulfate) 100 mg, I (as potassium iodide) 0.70 mg, Se (as sodium selenite) 0.40 mg; vitamin A, 10,000 IU; vitamin D, 3,000 IU; vitamin K_3_, 3 mg; thiamine, 5 mg; riboflavin, 10 mg; pyridoxine, 13 mg; cobalamin, 0.01; niacin, 50 mg; pantothenic acid, 17 mg; folic acid, 0.3 mg; biotin, 0.2 mg.
